# Combined Presentation of Acute Confusion and Severe Pancytopenia in Vitamin B12 Deficiency

**DOI:** 10.7759/cureus.40236

**Published:** 2023-06-10

**Authors:** Fatima Ghazal, Michelle Zur, Aaron Silver

**Affiliations:** 1 Internal Medicine, University of Connecticut School of Medicine, Farmington, USA; 2 Internal Medicine, University of Connecticut Health, Farmington, USA; 3 Hospital Medicine, Hartford Hospital, Hartford, USA

**Keywords:** pancytopenia, “pancytopenia”, confusion, neurological manifestations, hematological manifestations, vitamin b12

## Abstract

Vitamin B12 deficiency is more prevalent in the elderly and can develop as a result of malnutrition, malabsorption, chronic alcoholism, and chronic use of common medications (e.g. metformin, PPI, methotrexate) along with other causes. A wide spectrum of hematological and neuropsychiatric manifestations exist with the most common being Megaloblastic anemia and subacute combined degeneration, respectively. The mechanisms leading to the manifestations specific to these two organ systems are thought to be different. The severity of neuropsychiatric presentation is reported to be inversely proportional to that of hematological presentation, thus making it uncommon for both to be readily apparent simultaneously. Regardless of the severity of the clinical presentation, a good response to vitamin B12 replacement therapy is reported despite the lack of guidelines regarding dosing, frequency, or duration of treatment needed to note improvement in manifestations. The aim of this report is to increase the provider’s knowledge that a severe combined hematological and neuropsychiatry manifestation can co-exist and report the management used for recovery.

## Introduction

Vitamin B12, known as cobalamin, is available in dietary sources and binds to intrinsic factors secreted by gastric parietal cells to be later absorbed in the terminal ileum. It is stored in the liver with stores lasting one to three years prior to complete depletion after intake cessation [1]. Vitamin B12 deficiency causes are related to either decreased intake such as a strict vegetarian diet or malnutrition or decreased absorption that can be caused by pernicious anemia, chronic alcohol use, gastric surgery, and pancreatic insufficiency among many others [2]. The prevalence of vitamin B12 deficiency increases from 3-5% in the general population to 5-20% in the elderly [3], which can be explained by poly-medication and gastrointestinal diseases [4].

The most common hematological manifestation is megaloblastic anemia with elevated methylmalonic acid (MMA) and total homocysteine [5-7]. Nonetheless, although uncommon, life-threatening hematological presentations are reported, including pancytopenia and pseudo-thrombotic microangiopathy. Subacute combined degeneration of the spinal cord is the classical neurological clinical presentation of vitamin B12 deficiency, resulting in both motor and sensory deficits [4,8]. Other neurologic syndromes reported at a lower rate include optic neuropathy, Paresthesia, both with and without abnormal neurological findings, and myelopathy [9]. The most common psychiatric presentation is behavioral disturbance followed by progressive cognitive decline including dementia or delirium [2,8,10]. Other well-reported manifestations include paranoia, reversible psychosis, and depression [11]. It is notable that the mechanism leading to the hematological sequela of vitamin B12 deficiency is different than that leading to the neuropsychiatric manifestation, suggesting that the severity of both is inversely proportional [12].

Here, we describe a case of an elderly gentleman with a history of chronic alcohol use presenting in a combined state of acute confusion and severe pancytopenia attributed to vitamin B12 deficiency. Our goal is to report the rare manifestations of vitamin B12 deficiency with combined severity in both hematological and neurological aspects, as well as enrich the provider’s knowledge in regard to the management and timeline to recovery.

## Case presentation

A 78-year-old male patient presented to the hospital with a history of progressive fatigue, generalized weakness, confusion, and jaundice of unclear duration. His collateral history was obtained from family members due to his confusion; they reported that he lives alone and was independent in his activities of daily living, until around four to six weeks prior to presentation when he started reporting progressive fatigue and generalized weakness leading to difficulty walking. He was noted to be confused with jaundice on the day of his presentation, prompting his hospital transfer. His past medical history was unremarkable apart from chronic heavy alcohol use. The patient did not have any previous hospitalizations or regular physician office visits and was not taking any medications at the time. On examination, the patient was oriented only to self and irritable with poor concentration. He appeared pale with diffuse body jaundice and scleral icterus. The remaining dermatological examination was unremarkable. The neurological examination was limited, as the patient was uncooperative and in a state of confusion. Reflexes were symmetric and preserved. No abnormal findings were reported on respiratory, cardiac, or abdominal examination. The patient was afebrile with a blood pressure of 100/50 mmHg, pulse of 86 beats/min, and respiratory rate of 18 breaths/min.

**Figure 1:**
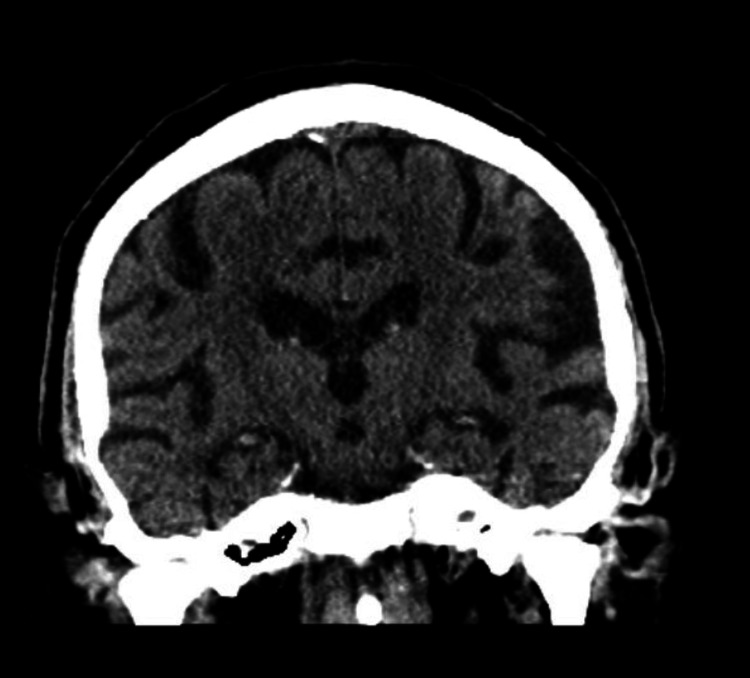
CT scan head without contrast Generalized cerebral volume loss

**Figure 2:**
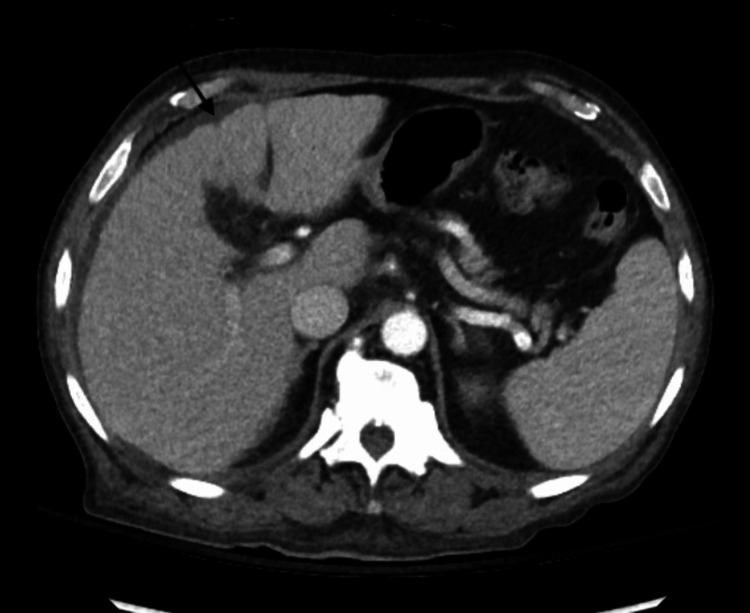
CT scan abdomen and pelvis with contrast Subtle nodular contour of the liver suggestive of cirrhosis

Complete blood count (Table 1) revealed pancytopenia, white blood cell count of 1.7 thousand/ul with an absolute neutrophil count of 900 uL, hemoglobin of 3.8 g/dl (confirmed twice), and platelet count of 25,000 uL. A peripheral blood smear showed macrocytes, tear drop cells, and occasional schistocytes. Elevated total bilirubin was 2.3 mg/dl with a direct bilirubin of 1 mg/dl, reticulocyte count of 2.4% with a low absolute count, low haptoglobin of <10 mg/dl, and elevated lactate dehydrogenase (LDH) of 1536 U/L. A normal folate level of 8 ng/ml and a low vitamin B12 level of < 150 pg/ml were reported. The patient’s iron studies, thyroid function tests, liver function tests, coagulation panels, electrolytes, and creatinine were within normal limits. The ethanol level was <11 mg/dl with normal ammonia and copper levels. HIV, syphilis, and coronavirus disease 2019 (COVID-19) serology testing were all negative with normal alpha-fetoprotein (AFP) tumor markers. However, the intrinsic factor antibody was positive. CT scan of the head without contrast revealed generalized cerebral volume loss (Figure 1). The CT scan of the abdomen and pelvis with contrast demonstrated a subtle nodular contour of the liver, suggestive of cirrhosis (Figure 2). The chest radiography was unremarkable.

**Table 1:** Laboratory values of anemia workup with relevant findings MCV: mean corpuscular volume; LDH: lactate dehydrogenase

Lab Workup	Value
White Blood Cell Count	1.5 x 103 ul
Hemoglobin	3.7 g/dl
Hematocrit	10.90%
Platelet Count	25 x 103 ul
MCV	131 fL
Folate	8 ng/ml
Vitamin B12	< 150 pg/ml
Haptoglobin	<10 mg/dl
Reticulocyte Count	2.40%
LDH	1536 U/L
Total Bilirubin	2.3 mg/dl

Severe pancytopenia with a hemolytic-like picture and acute confusion was attributed to vitamin B12 deficiency in the setting of pernicious anemia. He was transfused with three units of packed red blood cells to maintain a hemoglobin level of > 7 g/dl. The patient initially received 1000 mcg of cyanocobalamin intra-muscularly daily for a total of 7 days. Vitamin B12 levels improved to 2000 pg/ml and injections decreased to weekly for a total of four doses then monthly afterward. The platelet count started improving after five days of treatment and normalized after eight days. White blood cell count returned to a normal level with a resolution of neutropenia within one week of treatment. From a neuropsychiatric perspective, the patient's condition worsened during the first few days of hospitalization with confusion, agitation, and restlessness requiring geriatric medicine input for pharmacological management. Improvement in agitation was noted within 10 days of cyanocobalamin replacement, with persistent confusion.

The patient was eventually discharged to a skilled nursing facility with a plan to remain on monthly cyanocobalamin injections and GI outpatient referral for endoscopy in the future. At the three-month outpatient primary care follow-up visit, cognitive function significantly improved with no further details provided. No focal or neurological deficits later emerged. He remains off alcohol since his hospitalizations and is on monthly 1000 mcg cyanocobalamin injections for persistent mild anemia (the latest hemoglobin was reported to be 12.2 g/dl).

## Discussion

Vitamin B12 deficiency is common in the elderly population [7]. Despite that, it can go unrecognized in patients with multiple comorbidities [11]. Despite the fact that hematological and neuropsychiatric manifestations are well-known, life-threatening and rare presentations do exist and are not well-reported, leading to misdiagnosis. The patient described here had severe and atypical manifestations of vitamin B12 deficiency. His neuropsychiatric symptoms, including generalized weakness and progressive fatigue, are common and in the setting of chronic heavy alcohol drinking, raise concern for vitamin B12 deficiency. However, the acute confusion with severe agitation and restlessness requiring multiple anti-psychotic and anxiolytic medications was a severe presentation, in which more life-threatening differentials had to be ruled out first, which was done through the CT head. His hematological manifestations were also uncommon. In a study of 201 consecutive patients with documented cobalamin deficiency, 5% of the patients with hematological manifestations had severe symptomatic pancytopenia, 2.5% had severe anemia with a hemoglobin level <6 g/dl, and 1.5% had hemolytic anemia [13]. Our patient had a combination of severe pancytopenia with a hemoglobin level of 3.8 g/dl and hemolytic anemia.

Another rare aspect of the clinical presentation in the case described is the combination of a severe neuropsychiatric and hematological manifestation, as they occur through different mechanisms [4,12]. Vitamin B12 deficiency leads to desynchrony in the maturation of the erythrocyte cytoplasm and nuclei leading to megaloblastic anemia [13]. In severe cases, this can lead to dysplastic bone marrow and ineffective hematopoiesis, leading, in rare cases, to life-threatening anemia and pancytopenia [12-14]. This can be mistaken for acute leukemia [5]. On the other end, neurologically, vitamin B12 deficiency affects the initial myelination and maintenance of its normal function, leading to typical subacute combined degeneration [13]. The literature reports that the combination and severity of both hematological and neuropsychiatric manifestations are inversely proportional though the mechanism explaining this theory remains unclear [12,13].

In most cases, a vitamin B12 level of <200 pg/ml is sufficient to establish a diagnosis [11]. However, elevation in methylmalonic acid and homocysteine can increase the specificity and sensitivity of the diagnosis [9,10]. Delay in diagnosis can occur, as other disorders, such as spinal cord compression, malignancy, and peripheral neuropathy due to common comorbid conditions like diabetes, are usually ruled out prior to obtaining the vitamin B12 level, leading to the progression of the neurological manifestations [9]. However, due to the severe hematological presentation in our patient, the vitamin B12 level was obtained on admission, establishing the diagnosis within 24 hours and leading to early treatment. In addition to the heavy alcohol intake, the patient had a positive intrinsic factor antibody, making pernicious anemia an additional cause of vitamin B12 deficiency. Noting that pernicious anemia is the most common cause of vitamin B12 deficiency and must be considered even if other causes of vitamin B12 deficiency exist, as it affects treatment duration [4,6].

Different doses, routes, and frequencies of vitamin B12 supplementation have been reviewed. Although both oral and parenteral routes are reported to have equal efficacy in resolving both hematologic and neuropsychiatric manifestations, the parenteral route is the preferred method of administration, at least initially until the resolution of symptoms occurs [9,15]. Different doses of vitamin B12 supplementation have been used, ranging from 120 to 2000 mcg [7]. However, 1000 mcg has been more prevalent in the literature, with dosing initially daily until improvement occurs, then weekly for four to six weeks, and then monthly until complete resolution of all symptoms occurs [8,13]. This is very similar to the management in our case, as the patient received 1000 mg daily intra-muscular injections for seven days until his blood count improved. This was followed by weekly injections for four weeks before being transitioned to monthly injections. In patients with pernicious anemia, vitamin B12 supplementation is life-long, and it is responsive to oral formulas [8,9].

Case reports of combined severe hematological presentation are rare, however, they all mention a faster rate of hematological improvement than neurological, which is similar to the timeline in our patient. It has been reported that anemia starts improving within a week of vitamin B12 supplementation and achieves complete resolution within six to eight weeks while confusion can take several months [13]. The patient described in this case achieved improvement in regard to his anemia and resolution of thrombocytopenia and leukopenia by day eight. His agitation, restlessness, and confusion worsened over the first few days of treatment, requiring geriatric medicine input. Agitation later improved within a week while confusion required three months. More research is needed to guide the dose, frequency, and route of vitamin B12 administration, as not enough studies exist to identify their effect on the recovery period. 

## Conclusions

Vitamin B12 deficiency is a common condition that increases in incidence with age. Given that vitamin B12 functions on multiple organ systems in different mechanisms of action. This can lead to a variety of clinical manifestations that rarely co-exist. A thorough history and physical examination with a high level of clinical suspicion are warranted to avoid misdiagnosis. Vitamin B12 supplementation does help with symptom resolution, although the time frame varies with the route and dose of supplementation in addition to the initial organ system affected.
